# Awareness Among Undergraduate Students of Mangalore City Regarding Novel Coronavirus (COVID-19): A Questionnaire Study

**DOI:** 10.1017/dmp.2020.204

**Published:** 2020-06-24

**Authors:** Deeksha Das, Ramya Shenoy, Megha Mukherjee, Bhaskaran Unnikrishnan, Nikita Rungta

**Affiliations:** Manipal College of Dental Sciences, Mangalore, Manipal Academy of Higher Education, Manipal, Karnataka, India; Department of Public Health Dentistry, Manipal College of Dental Sciences, Mangalore, Manipal Academy of Higher Education, Manipal, Karnataka, India; Manipal College of Dental Sciences, Mangalore, Manipal Academy of Higher Education, Manipal, Karnataka, India; Department of Community Medicine, Kasturba Medical College, Mangalore, Manipal Academy of Higher Education, Manipal, Karnataka, India; Manipal College of Dental Sciences, Mangalore, Manipal Academy of Higher Education, Manipal, Karnataka, India

**Keywords:** COVID-19, epidemic, hand washing, pandemic, young adults

## Abstract

**Objectives:**

COVID-19 outbreak has surfaced as an imminent threat for the public health. Because India is a populous country, it is important for Indians to be aware of the basic modes of prevention that can diminish the spread of the coronavirus disease 2019 (COVID-19) infection.

**Aim:**

The present questionnaire study was carried out among the undergraduate students to assess the awareness regarding the spread and control of COVID-19.

**Methods:**

The questionnaire was circulated among the undergraduate students as a Google form.

**Results:**

The study included responses of 868 undergraduate students belonging to 2 university colleges. The majority of the participants were females (63%; *n* = 547) in the age range of 18-23 y. Approximately 98.3% (853) had awareness regarding COVID-19. Approximately 94.7% (822) were washing their hands after visiting public places, out of which only 90.6% (786) were aware of proper steps to be followed in hand washing. It was concluded that it is required to create awareness among 20.8% (181) of our study participants regarding the importance of hand washing to control COVID-19.

**Conclusions:**

Awareness regarding COVID-19 among study participants was good. However, a small part of the study population is required to be educated on proper steps to be followed in hand washing.

A series of cases of a novel virus causing respiratory infections in humans was observed in people of Wuhan, China, in December 2019. The disease caused by this novel virus was named as coronavirus disease 2019 (COVID-2019), This virus was isolated on January 7, 2020. It was morphologically similar to the virus that causes severe acute respiratory syndrome, or popularly known as SARS. However, the COVID-19 outbreak has surfaced as an imminent threat for the public health and has posed as a critical challenge for research and medical communities.^1^

Broad steps to minimize human-to-human transmission of COVID-19 is required to control the current outbreak. Special efforts and attention to protect or decrease transmission should be applied in populations with high susceptibilities, such as health-care providers and elderly people. The early death cases of COVID-19 outbreak occurred in the elderly due to a compromised immune system that allows for a rapid progression of viral infection. The public services and facilities should provide decontaminating reagents for cleaning hands on a routine basis. Physical contact with wet and contaminated objects should be dealt cautiously, especially agents such as fecal and urine samples that can potentially serve as an alternative route of transmission. Epidemiological changes in COVID-19 infection should be monitored, keeping in mind the potential routes of transmission and subclinical infection, along with the adaptation, evolution, and virus spread among humans and possible intermediate animals and reservoirs. There remains a considerable number of questions that need to be addressed.^2^

Various bodies including the World Health Organization (WHO) and Centers for Disease Control and Prevention (CDC) have issued advice on preventing further spread of COVID-19. They recommend avoiding travel to high-risk areas and contact with individuals who are symptomatic from regions with known COVID-19 outbreak. Basic hand hygiene measures are also recommended, including frequent hand washing and the use of personal protective equipment (PPE), such as face masks for people showing symptoms. It is also our responsibility to be aware of the signs and symptoms and to promptly address them.^3^ Comprehensive protective measures, such as face mask and hand hygiene are included in public health guidelines for pandemic preparedness. Most coronaviruses, including COVID-19, can be inactivated by alcohol-based hand sanitizers and disinfectants, such as bleach. Environmental disinfection using appropriate sanitizers is also recommended. Because hand hygiene does not affect the direct transmission of COVID-19 by respiratory droplets, face masks have been widely used by at-risk populations in China and some other locations in Asia, such as Taiwan and Hong Kong. The efficacy of face masks among healthy individuals is questionable, but masks can protect others, particularly health-care workers, from active symptomatic individuals with COVID-19. The combination of masks and hand hygiene, however, has been shown to reduce transmission of respiratory viruses. Face mask use should be recommended for infected persons, for uninfected persons who are caring for infected persons, and for those interacting in extensive crowded settings where widespread community transmission is possibly occurring.^4^

In India, it has been found that some patients fled the hospital wards when they were asked to stay under quarantine. This comes as no surprise as health facilities in India are not as efficient as they are expected to be to fight the rapid spread of COVID-19. As per World Bank, India only spends 1.28% of the gross domestic product on health facilities. This underdeveloped infrastructure of health could be catastrophic at times of health emergencies, such as COVID-19.^5^

Hence, the people of India need to be aware of the basic modes of prevention and other necessary precautionary measures that can diminish the spread of the infection. Our present study was carried out among the undergraduate students of Mangalore to assess the awareness regarding spread and control of this novel malady.

## METHODS

The study included 868 undergraduate college students belonging to 2 university colleges in Mangalore in coastal South India from varying socio-economic status. A lottery method was used to select these 2 colleges of 7 university colleges of Mangalore city. Prior permission from Principal of the concerned colleges were taken. Young adults in the age group of 18-23 y were included in the study sample.

Before commencement of the study, clearance from the Institutional Ethics Committee was obtained, and consent from each person was documented to ensure their voluntary and active participation in the study (Protocol No. 20030).

### Sample Size

The sample size for a finite population considering 5% of 4600 population, confidence level 99 and confidence interval 0.01.
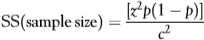


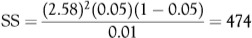



The questionnaire consisted of 12 questions regarding COVID-19 and was prepared by the authors. The content and construct validity were checked with 4 subject experts. The questionnaire (Table 1) was circulated by means of Google docs among the study participants, and the data were collected during a 1-wk period. The Google doc had 2 parts. In the first part, participant had to consent only then they could access the second part of questions. The link was sent to 500 undergraduate students belonging to 2 university colleges of Mangalore in coastal South India. A total of 868 students responded and filled out the questionnaire. This may be due to the sharing of the link among their friends. All the 868 responses were considered in this study. The data obtained from the study were compiled and statistically analyzed using SPSS software version 20. Upon completion and submission of the questionnaire, relevant information, especially on the importance of hand washing, wearing masks, and social distancing to prevent COVID-19 infection, was sent to the volunteers by means of e-mail.

**TABLE 1:** Participants Responses to the Questions Regarding COVID-19

	Questions	Responses		
		Yes	No	Not Answered
1.	Are you aware of the novel coronavirus epidemic?	853 (98.3%)	15(1.7%)	
2	If yes, through which media did you get to know about it?			
	Social media	761(8.7%		
	Television	456(53.1%)		
	Friends/families	439(51.2%)		
	Hospitals	130(15.2%)		
	Newspapers	354(41.3%)		
	Caller tunes	2(0.2%)		
3	Are you aware of the symptoms related to the disease?	829(95.5%)	31(3.6%)	8(0.9%)
4	Are you aware how the infection spreads?	832(95.8%)	33(3.8%)	3(0.3%)
5	Do you know whom you need to contact in case you suspect any of the symptoms?	728(83.8%)	131(15.0%)	9(1.0%)
6	If you suspect any flu-like symptoms would you go for a test?	749(86.2%)	101(1.6%)	8(0.9%)
7	Would you avoid contact with pets or animals if you are sick?	688(79.4%)	164(18.9%)	6(0.7%)
8	Do you avoid visiting hospitals from the fear of mass panic/suspicion of getting infected or testing positive?	612(70.5%)	239(27.5%)	17(2.0%)
9	Do you know about the basic precautions to be taken to combat the spread?	781(89.9%)	74(8.5%)	13(1.5%)
10	Do you wash your hands regularly?	808(93.0%)	48(5.5%)	12(1.4%)
11	Do you wash your hands after coming from a public place such as hospital/school/workplace?	822(94.7%)	34(3.9%)	2(0.4%)
12	Are you aware of the steps of hand washing?	786(90.6%)	71(8.2%)	1(0.2%)

## RESULTS

A total of 868 undergraduate college students of Mangalore in coastal South India participated in this study. The majority of individuals were females (63%; *n* = 547) in the age range of 18-23 y (mean age, 20.75 ± 1.80 y). All the responses from the respondents were compiled and are depicted in Table 1.

Approximately 98.3% (853) had awareness regarding COVID-19, and the majority of them got to know about it through social media 88.7% (761), followed by television 53.1% (456), friends/families 51.2% (439), and newspaper 41.3% (354). The government has recently introduced caller tunes on coronavirus awareness. It was shown through this survey that only 2 students got to know about this virus through caller tunes.

Regarding symptoms related to COVID-19 and how the infection spreads, a total of 95.5% (829) of students were aware of that. However, only 83.8% (728) knew who they should contact if they suspect infection. Approximately 86.2% (749) were ready to get a test done if they have flu-like symptoms, and 79.4% (688) would avoid contact with animals. It was also revealed from this survey that 89.9% (781) knew about the basic precautions to be taken to prevent this disease from spreading.

When the analysis was done to assess the importance of hand washing to control this infection, it was noted that 93% (808) had the habit of washing their hands regularly. Approximately 94.7% (822) were washing their hands after visiting public places. But only 90.6% (786) were aware of proper steps to be followed during hand washing. Therefore, it is required to create awareness among 20.8% (181) of our study participants regarding the importance of hand washing to control COVID-19 spread.

## DISCUSSION

A similar study was conducted previously in Hong Kong to evaluate the knowledge and attitude of dental patients toward SARS during the SARS outbreak.^6^ Kharma et al.^7^ carried out a study where they tried to assess the knowledge of dental students toward MERS, and Ashok et al.^8^ carried out a study to assess the knowledge and apprehension of dental patients regarding MERS. To the best of our knowledge, this is the first time a questionnaire survey has been conducted to assess the awareness of novel coronavirus (COVID-19).

It has been advised by WHO that every person should follow hygiene measures, such as regular hand washing and avoid touching mouth, nose, or eyes with hands and avoid contact with sick people and animals.^3^ Whenever a person visits the hospital, it is mandatory to reveal their travel history. Persons with symptoms, such as cough, fever, and sore throat, should be instructed to cover his/her mouth with a tissue when coughing or sneezing, perform hand hygiene after contacting contaminated materials or respiratory secretions, and dispose of the used tissue in the waste receptacle. Masks should be offered to persons who are coughing, and the people who are coughing should be encouraged to sit at least 1 meter away from others in the waiting room. Overcrowding should be avoided as far as possible in clinical areas to prevent cross-infection. Standard procedures should be followed in the waste disposal and cleaning and disinfection of environmental surface and patient-care equipment.^9^ Our study participants showed good knowledge regarding hand hygiene and precautions to be taken in case of the presence of flu-like symptoms.

The main limitation of this study was that most of the questions included in the questionnaire were related to awareness and hand hygiene, and there were fewer questions related to infection control measures, such as wearing masks and the importance of social distancing. The study population was restricted to university students without a health science background. It would have been better if we had compared the present results with university students with a science background. Also, comparison between university students belonging to public and private universities and young professionals of the same age would have given a clear understanding of the result. Further investigation on diverse populations on knowledge and awareness of COVID-19 and its spread should be carried out.

## CONCLUSIONS

From the present study, it can be concluded that awareness regarding COVID-19 among study participants was good. Most of them were aware of the precautionary measures to be followed during the outbreak, but a small part of the study population requires education on proper steps to be followed in hand washing.
